# Supplementary material for: Clinical and Genetic Characterization of Patients with Bartter and Gitelman Syndrome
Source: Int J Mol Sci. 2022 May 18;23(10):5641. doi: 10.3390/ijms23105641 (PMC9144947; doi:10.3390/ijms23105641)
Supplement: Supplementary file 1 [file ijms-23-05641-s001.zip › Palazzo et al_Supplementary.pdf]

Supplementary Materials

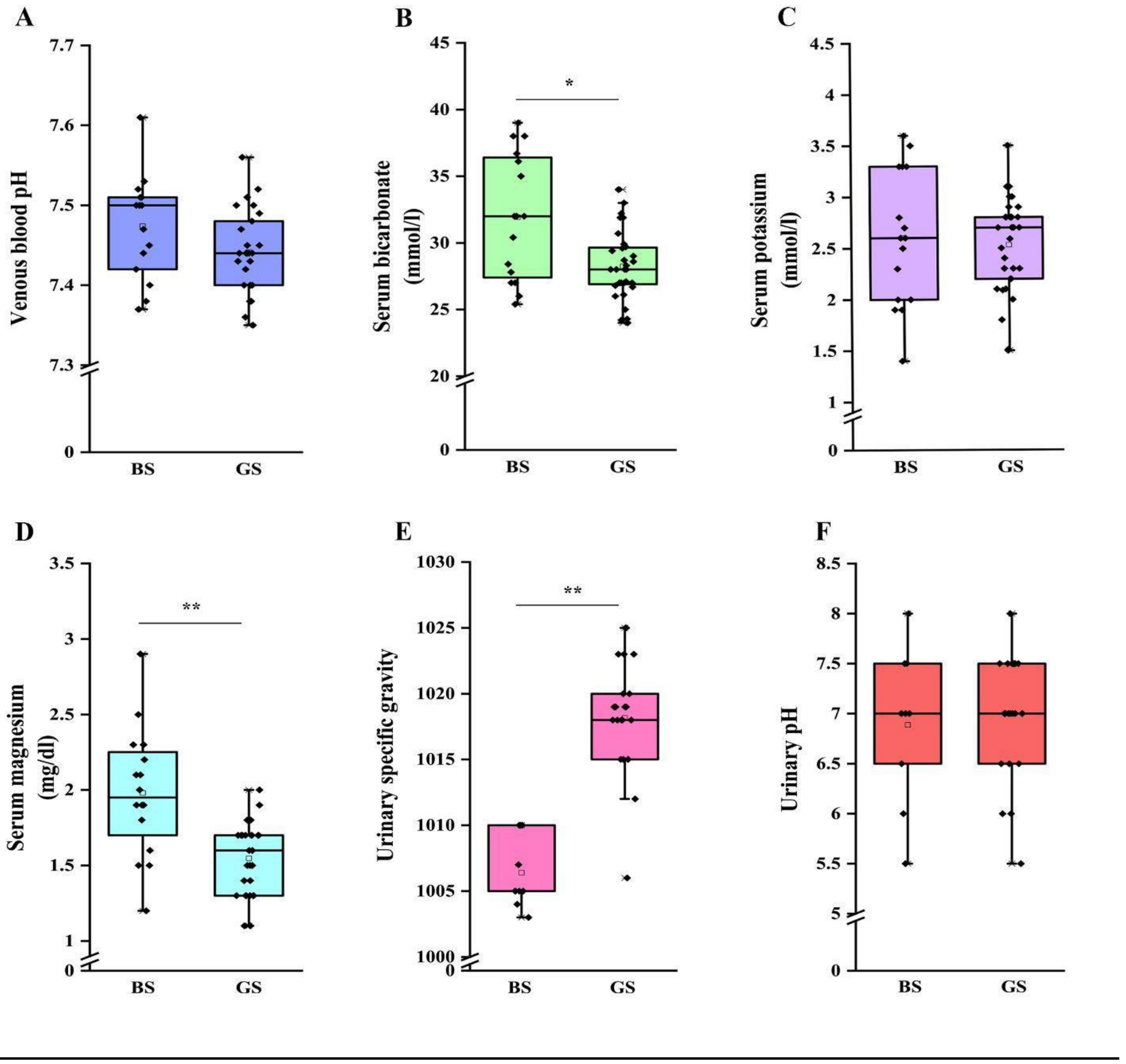

Figure S1. Laboratory features of patients with a molecular diagnosis of BS and GS. (A)Venous blood pH; (B) Serum bicarbonate levels (mmol/l); (C) Serum potassium levels (mmol/l); (D) Serum magnesium levels (mg/dl); (E) Urine specific gravity; (F) Urinary pH.

BS, Bartter syndrome; GS, Gitelman syndrome.

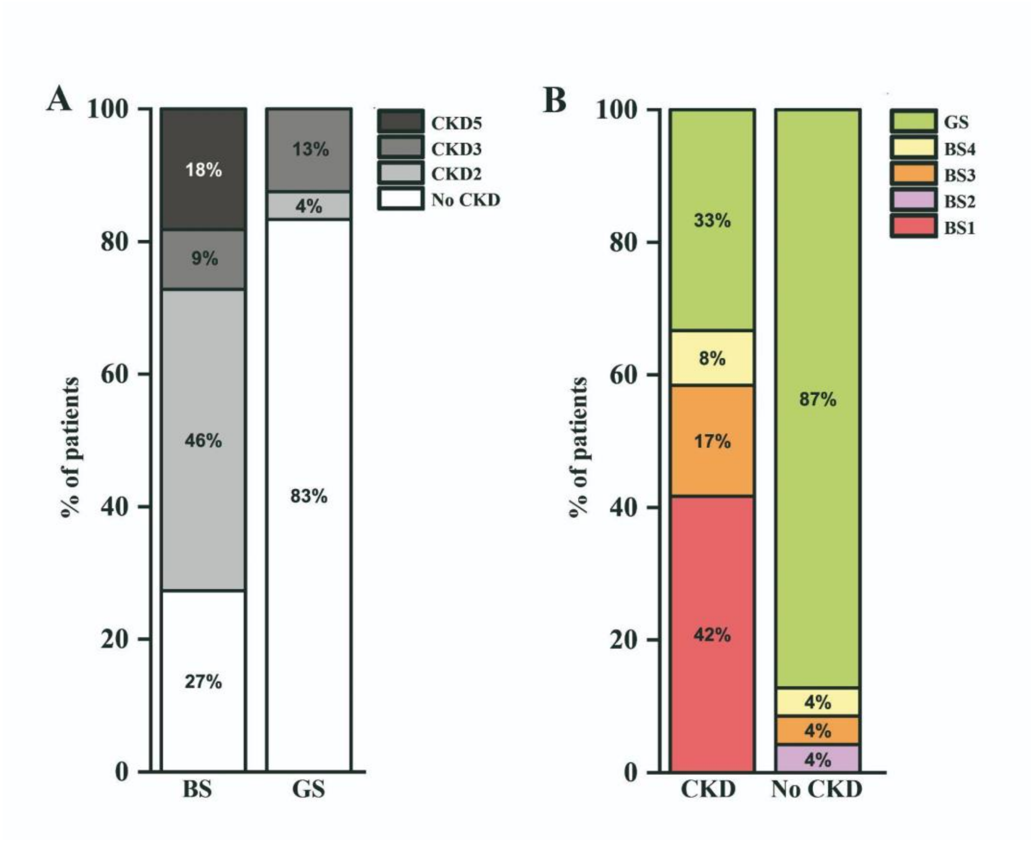

Figure S2. Frequency of CKD at last follow up in patients with a molecular diagnosis of BS and GS. (A) Frequency of different stages of CKD in patients with a molecular diagnosis of BS and GS. (B) Frequency of genetic diagnosis of BS1, BS2, BS3, BS4 and GS in patients with and without CKD at last follow up.

BS, Bartter syndrome; GS, Gitelman syndrome; BS1, Bartter syndrome type 1; BS2, Bartter syndrome type 2; BS3, Bartter syndrome type 3; BS4, Bartter syndrome type 4 (4a or 4b); CKD, chronic kidney disease; CKD2, CKD stage G2; CKD3, CKD stage G3; CKD5, end-stage kidney disease.

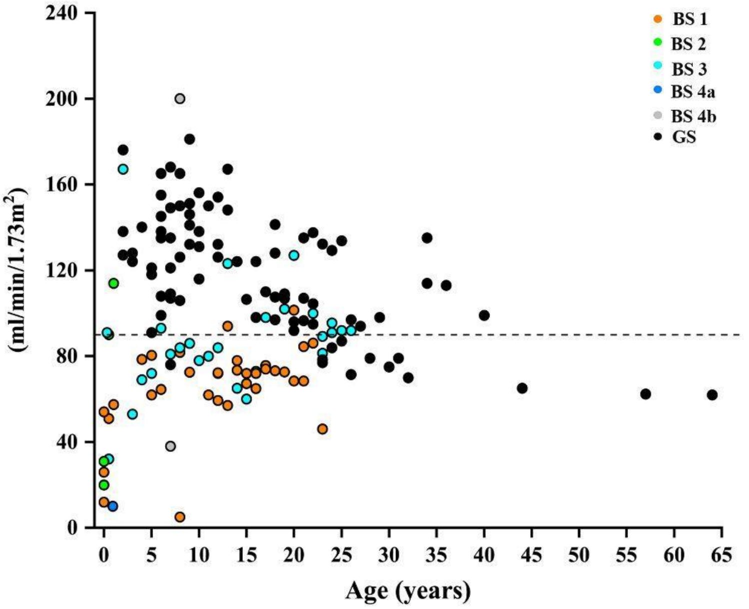

Figure S3. eGFR over time according to the genetic diagnosis. Plot of eGFR over time in patients with a molecular diagnosis of BS1 (orange dots), BS2 (green dots), BS3 (light blue dots), BS4a (blue dots), BS4b (grey dots) and GS (black dots).

BS, Bartter syndrome; GS, Gitelman syndrome; BS1, Bartter syndrome type 1; BS2, Bartter syndrome type 2; BS3, Bartter syndrome type 3; BS4a, Bartter syndrome type 4a; BS4b, Bartter syndrome type 4b; eGFR, glomerular filtration rate.

Table S1. Biochemical features of the patients included in the study.

Patient	pH (7.35 - 7.45)	HCO3 (17-29 mmol/l)	K (3.4 - 5.2 mmol/l)	Na (135-145 mmol/l)	Cl (101– 110 mmol/l)	Mg (1.41 - 1.85 mg/dl)	Ca (9.6-10.6 mg/dl)	sCr (mg/dl)	eGFR (ml/min/1.73m2)	Increased Aldosteron	Increased Renin	Urinary pH (5.5 - 7.5)	Urinary specific gravity (1010-1030)
Case 1	7.44	27	3.6	133	96	2.1	10.6	1.56	12	YES	YES	5.5	1007
Case 2	7.42	30.4	2.3	132	92	2.5	11.1	0.7	26	YES	YES	7.5	1010
Case 3	7.45	32	2	133	94	1.8	9.7	0.9	72	YES	YES	6.5	1004
Case 4	7.37	27.8	3.3	141	97	2.1	9.7	NA	NA	YES	YES	NA	NA
Case 5	7.5	25.4	3.3	124	92	2.9	4	0.96	20	YES	YES	7.5	1010
Case 6	7.47	38	2.7	138	85	1.6	9.5	0.65	31	YES	YES	NA	NA
Case 7	7.51	36.7	2.5	138	90	1.9	7	NA	NA	NO	NA	NA	1005
Case 8	7.61	35	1.4	130	56	1.2	9.2	0.7	32	YES	YES	7	1005
Case 9	7.53	36.1	2.6	138	93	2.3	10.4	0.37	91	YES	YES	8	1005
Case 10	7.51	38	1.9	130	84	1.5	7	NA	NA	YES	YES	NA	NA
Case 11	7.38	28.4	3.3	138	99	2.3	10.2	1.2	38	YES	YES	7	1003
Case 12	NA	NA	NA	NA	NA	NA	NA	NA	NA	NA	NA	NA	NA
Case 13	7.45	28.6	2.9	135	94	2	9.4	0.5	91	YES	YES	7	NA
Case 14	7.38	28.7	2.8	135	NA	1.4	9.62	0.4	135	NO	NO	NA	NA
Case 15	7.52	39	2.8	139	84	1.9	11.6	0.4	83	YES	YES	7	1010

**Case 16**	7.4	26	3.5	142	102	2.2	9.8	0.7	62	YES	YES	6	1005
**Case 17**	7.59	41.3	2.5	135	81	2	9.6	0.24	NA	YES	YES	7	1008
**Case 18**	7.45	26	3	141	98	1.7	NA	NA	NA	NA	NA	7	1023
**Case 19**	7.44	24.2	2.7	142	98	1.8	9.3	0.7	NA	YES	YES	NA	NA
**Case 20**	7.38	25	2.1	135	88	1.7	10.3	0.24	138	YES	YES	7.5	1015
**Case 21**	7.41	25.5	3.8	137	98	1.3	6	0.65	46	NO	NO	7	1007
**Case 22**	7.5	27.4	2.6	131	94	2.2	10.3	0.28	62	YES	YES	5.5	1005
**Case 23**	7.5	32	2	129	90	2	9.6	NA	NA	NA	NA	NA	NA
**Case 24**	NA	27	1.9	131	NA	1.9	8.07	0.68	31	NA	YES	NA	NA
**Case 25**	7.41	26	2.5	136	NA	0.9	9	0.6	112	NO	NO	NA	1007
**Case 26**	7.51	32.2	2.09	138	85	1.5	8.6	0.25	127	YES	YES	7.5	1020
**Case 27**	7.44	31.9	2.8	137	90	1.3	8.8	0.3	145	NA	NA	6	1025
**Case 28**	NA	NA	NA	NA	NA	NA	NA	NA	NA	NA	NA	NA	NA
**Case 29**	7.44	31.9	2.7	137	100	1.6	9.6	0.5	107	NO	NO	7	1018
**Case 30**	7.35	27	3.5	140	101	1.1	9.4	1	35	NO	NA	5.5	1015
**Case 31**	7.49	28.3	2.8	136	101	1.6	NA	NA	NA	YES	YES	NA	NA
**Case 32**	7.43	28	2.5	NA	99.2	1.3	NA	NA	NA	YES	YES	6.5	1019
**Case 33**	7.57	38.7	1.6	138	95	1.1	10	1.04	104	NO	NO	6	NA
**Case 34**	7.44	26.3	2.7	134	104	1.2	8.9	0.73	116	YES	YES	NA	NA

**Case 35**	7.44	29	1.5	129	100	2	9.7	NA	NA	YES	NO	NA	NA
**Case 36**	NA	28	1.51	142	95	NA	10.1	NA	NA	NO	NO	NA	NA
**Case 37**	NA	27	2.1	137	95	1.7	9	1.35	71.4	YES	YES	7.5	1012
**Case 38**	7.47	29.4	2.8	143	98	1.7	9.4	NA	NA	YES	YES	NA	NA
**Case 39**	NA	27	3.1	137	98	1.7	9.7	0.7	NA	NO	YES	NA	NA
**Case 40**	7.4	27.1	2.7	137	97	1.7	9.6	0.4	113	NO	NO	NA	1019
**Case 41**	7.4	24	2.7	133	93	1.5	9.6	0.27	130	YES	YES	7	1018
**Case 42**	7.5	28	2	140	98	1.3	11	NA	NA	NO	NO	NA	NA
**Case 43**	7.56	27	1.5	137	105	1.5	8.5	0.45	130	NA	NA	7	1015
**Case 44**	NA	27	2.8	NA	NA	1.8	NA	NA	NA	NA	NA	NA	NA
**Case 45**	7.52	29	2.8	136	90	1.3	10.4	0.66	76	NO	YES	7.5	1006
**Case 46**	7.43	29.9	2.3	133	88	1.9	9.5	0.33	166	NO	NO	8	1020
**Case 47**	7.36	24.3	3	136	98	1.3	10	0.55	116	NA	NA	6.5	1018
**Case 48**	7.48	28	2.3	140	NA	1.3	10.4	0.83	114	YES	YES	NA	NA
**Case 49**	7.5	30.7	2.2	141	102	1.8	9.7	0.4	143	NO	NO	7.5	1019
**Case 50**	7.52	43	2.8	131	91	2	10.7	0.62	120	NO	NO	NA	1008
**Case 51**	NA	28.5	2.6	130	NA	1.9	9.5	1.13	56.6	YES	YES	NA	NA
**Case 52**	NA	NA	NA	NA	NA	NA	NA	NA	NA	NA	NA	NA	1025
**Case 53**	NA	33	2.9	141	NA	NA	9.9	0.6	105.5	NA	NA	6	1015

**Case 54**	NA	34	2.2	NA	NA	NA	NA	0.57	NA	NA	NA	7	1018
**Case 55**	7.4	26.7	3.1	139	101	1.4	9.4	0.54	99	NA	NA	6.5	1023
**Case 56**	7.4	29.7	1.8	133	92	1.5	9.8	0.37	121	YES	YES	7	1023
**Case 57**	7.5	32	2.6	NA	NA	1.5	NA	NA	NA	NO	NA	NA	NA
**Case 58**	7.44	29.6	2.3	137	97	1.1	9.8	0.62	98	YES	YES	7.5	1019
**Case 59**	7.45	29.7	2.9	138	98	1.4	6.6	0.7	109	YES	YES	7	1008
**Case 60**	7.4	28	2.7	NA	NA	2	NA	NA	NA	NO	NO	NA	NA
**Case 61**	7.44	26.1	2.59	140	100	1.7	10.2	0.3	155	YES	YES	7.5	1018
**Case 62**	7.42	26.8	2.4	143	101	1.7	9.7	0.2	176	NO	NA	7.5	1015
**Case 63**	7.5	27	1.9	134	NA	NA	9.2	0.66	125	NO	NA	NA	NA

NA, not available; eGFR, estimated glomerular filtration rate; sCr, serum creatinine.

Table S2. Clinical features of the patients included in the study.

Patient	Sex	Ethnicity	Consanguinity	Family history	Polidramnios	Gestational Age (weeks)	Birth weight (grams)	Failure to thrive	Poliuria	Nephrocalcinosis	Hypercalciuria	SNHL
**Case 1**	F	Caucasian	NO	NO	YES	28	1128	YES	YES	YES	YES	NO
**Case 2**	F	Caucasian	NO	NO	YES	29+4	1100	YES	YES	NO	YES	NO
**Case 3**	M	Caucasian	NO	YES	YES	36	3270	NO	NA	YES	YES	NO
**Case 4**	F	Caucasian	NO	NO	YES	24	550	YES	YES	YES	YES	NO
**Case 5**	F	Caucasian	NO	NO	YES	35+5	2010	YES	NA	YES	NO	NO
**Case 6**	M	Caucasian	NO	NO	YES	36	2590	NO	YES	NO	NA	NO
**Case 7**	M	South Asian	YES	YES	NO	38	3685	NO	NO	NO	NO	NO
**Case 8**	F	Caucasian	NO	YES	YES	38	3500	YES	NA	NO	YES	NO
**Case 9**	F	Caucasian	NO	NO	NO	38	3150	YES	NA	NO	NA	NO
**Case 10**	F	Caucasian	NO	NO	YES	26 +6	750	YES	YES	NO	NA	YES
**Case 11**	F	Caucasian	NO	YES	YES	36	1990	YES	NA	YES	NO	NO
**Case 12**	F	Caucasian	NA	NA	NO	NA	NA	NO	NA	NO	NA	NO
**Case 13**	M	Caucasian	NO	NO	NO	NA	2890	YES	NA	YES	NO	NA
**Case 14**	F	Caucasian	NO	NO	NO	NA	NA	YES	NO	YES	NO	NO
**Case 15**	M	Caucasian	NO	NO	NO	40	3100	YES	NO	NO	NO	NO
**Case 16**	F	Caucasian	NO	NO	YES	34+6	2070	NO	YES	YES	NO	NO

**Case 17**	F	Caucasian	NO	NO	NO	39+5	3045	YES	NO	NO	NO	NO
**Case 18**	M	Caucasian	NO	NO	YES	39	3300	YES	NO	NO	NO	NO
**Case 19**	M	Caucasian	NA	NA	NO	NA	NA	NO	NA	NO	NA	NO
**Case 20**	F	Chinese	NO	YES	NO	39+5	3168	YES	NA	NO	NO	NO
**Case 21**	F	Caucasian	NO	NO	NO	41+4	4175	NO	NA	YES	YES	NO
**Case 22**	F	Caucasian	NO	NO	YES	33+3	1836	NO	NA	YES	YES	NO
**Case 23**	F	Caucasian	NO	YES	YES	32	1200	YES	YES	YES	NA	NO
**Case 24**	M	Caucasian	NO	NO	YES	23+4	1750	NA	NA	YES	YES	NO
**Case 25**	F	Caucasian	NO	YES	NA	NA	NA	NO	NA	NO	NA	NO
**Case 26**	M	Caucasian	NO	YES	NO	38	3470	YES	NO	NO	NO	NO
**Case 27**	M	Caucasian	NO	YES	NO	41	3870	NO	NA	NO	NO	NO
**Case 28**	M	Caucasian	NO	NO	NA	NA	NA	NO	NO	NA	NA	NA
**Case 29**	F	Caucasian	NO	NO	NO	38	3220	NO	NA	NO	NO	NO
**Case 30**	M	Caucasian	NO	NO	NO	39	3450	NO	NA	YES	NO	NO
**Case 31**	M	Caucasian	NA	NA	NO	NA	NA	NO	NA	NO	NA	NA
**Case 32**	M	Caucasian	NO	NO	NO	39+4	NA	NO	NA	NO	NA	NA
**Case 33**	M	Caucasian	NO	NO	NO	39	3250	NO	NO	NO	NO	NO
**Case 34**	F	Caucasian	NO	NA	NO	NA	NA	NO	NA	YES	NO	NO
**Case 35**	F	Caucasian	NO	NO	NO	NA	NA	NO	YES	NO	NO	NO

**Case 36**	M	Caucasian	NO	NO	NO	NA	NA	NO	YES	NO	NA	NO
**Case 37**	M	Caucasian	NO	NO	NO	NA	NA	NO	NA	NO	NO	NO
**Case 38**	M	Indian	NA	NA	NO	NA	NA	NO	NA	NO	NA	NO
**Case 39**	M	Caucasian	NA	NA	NO	NA	NA	NO	NA	NO	NA	NO
**Cases 40**	M	Caucasian	NO	YES	NO	36+6	3570	YES	NA	NO	NO	NO
**Cases 41**	M	Caucasian	NO	YES	NO	39	3060	YES	NO	NO	NO	NO
**Cases 42**	F	Caucasian	NO	YES	NO	NA	NA	NO	NA	NO	NO	NO
**Case 43**	M	Caucasian	NO	NO	NO	37	2410	YES	NA	NO	NO	NO
**Case 44**	M	Caucasian	NO	NO	NA	NA	NA	NO	NA	NO	NA	NO
**Case 45**	M	Caucasian	NO	NO	NO	41+3	3650	YES	NA	NO	NO	NO
**Case 46**	F	Caucasian	NO	YES	NO	40+2	3390	NO	NO	NO	NO	NO
**Case 47**	F	Caucasiano	NO	NO	NO	40	3350	NO	NA	NO	NO	NO
**Case 48**	M	Caucasian	NO	NO	NO	NA	NA	NO	NO	NO	NO	NO
**Case 49**	F	East Asian	NO	NO	NO	NA	NA	NO	NA	NO	NO	NO
**Case 50**	F	Caucasian	NO	NO	NO	42	NA	NO	NA	NO	NO	NO
**Case 51**	F	Caucasian	NA	NA	NO	NA	NA	NO	NA	NO	NO	NO
**Case 52**	M	Caucasian	NO	NO	NA	NA	NA	NO	NO	NA	NA	NO
**Case 53**	F	Caucasian	NO	YES	NA	NA	NA	NO	NA	NO	NA	NA
**Case 54**	F	Caucasian	NO	YES	NA	NA	NA	NO	NA	NO	NA	NO

**Case 55**	M	Caucasian	NO	YES	NO	NA	NA	NO	NO	YES	NO	NO
**Case 56**	M	Caucasian	NO	YES	NO	39+1	3320	NO	NO	YES	NO	NO
**Case 57**	F	Caucasian	NO	NO	NO	NA	NA	NO	NA	NO	NA	NO
**Case 58**	M	Caucasian	NO	NO	NO	42	4200	YES	NO	NO	NO	NO
**Case 59**	F	Caucasian	NO	NO	NO	NA	NA	NO	NA	NO	YES	NO
**Case 60**	M	Caucasian	NO	YES	NO	NA	NA	NO	NA	NO	NA	NO
**Case 61**	F	Caucasian	NO	YES	NO	41	3890	YES	NO	NO	NO	NO
**Case 62**	F	Caucasian	NO	NO	NO	39	3600	YES	NO	NO	NO	NO
**Case 63**	F	Caucasian	NO	NO	NO	NA	NA	NO	YES	NO	NA	NO

M, male; F, female; NA, not available; SNHL, sensorineural hearing loss.

Table S3. Genetic findings.

Patient	GENE	Nucleotide change	Aminoacid change	Inheritance	Functional Impact	Ref	ACMG score Variant class
**Case 1**	*SLC12A1*	c.1041_1045delTCCAT<br>c.1041_1045delTCCAT	p.Pro348Glnfs*3<br>p.Pro348Glnfs*3	mat<br>pat	CL<br>CL	[1]<br>[1]	P<br>P
**Case 2**	*SLC12A1*	c.548T>A<br>c.820A>G	p.Val183Glu<br>p.Met274Val	pat<br>mat	PL<br>PL	ND<br>ND	LP<br>LP
**Case 3**	*SLC12A1*	c.1139T>G<br>c.1630C>T	p.Phe380Cys<br>p.Pro544Ser	NA<br>NA	PL<br>PL	[2]<br>ND	LP<br>LP
**Case 4**	*SLC12A1*	c.3164+1G>A<br>c.1190G>C	p.splicing alteration<br>p.Gly397Ala	pat<br>NA	CL<br>PL	[3]<br>[2]	P<br>LP
**Case 5**	*KCNJ1*	c.122G>A<br>c.808C>T	p.Arg41Lys<br>p.His270Tyr	pat<br>mat	PL<br>PL	ND<br>[3]	LP<br>LP

**Case 6**	*CLCNKB*	1p36.13(16.364.253-16.383.299)<br>1p36.13(16.364.253-16.383.299)	entire gene deletion<br>entire gene deletion	mat<br>pat	CL<br>CL	[4]<br>[4]	P<br>P
**Case 7**	*CLCNKB*	c.910C>T<br>c.910C>T	p.Arg304*<br>p.Arg304*	mat<br>pat	CL<br>CL	ND<br>ND	P<br>P
**Case 8**	*CLCNKB*	c.371C>T<br>c.1393G>A<br>1p36.13(16.364.253-16.383.299)x1	p.Pro124Leu<br>p.Gly465Arg<br>entire gene deletion	pat<br>pat<br>mat	PL<br>PL<br>CL	[4]<br>[5]<br>[4]	LP<br>LP<br>P
**Case 9**	*CLCNKB/CLCNKA*	c.2016+1G>A<br>1p36.13(16,353,805- 16,375,077)x1	p.spicing alteration<br>exon 8-20 *CLCNKA*-exon1-7 *CLCNKB* del	NA<br>NA	CL<br>CL	ND<br>ND	P<br>P
**Case 10**	*BSND*	c.272+1G>T<br>c.272+1G>T	p.splicing alteration<br>p.splicing alteration	mat<br>NA	CL<br>CL	ND<br>ND	P<br>P
**Case 11**	*CLCNKB/CLCNKA*	1p36.13(16353804 -16372181)<br>1p36.13(16353804 -16372181)	exon 6-20 *CLCNKA*- exon 1-3 *CLCNKB* del<br>exon 6-20 *CLCNKA*- exon 1-3 *CLCNKB* del	NA<br>NA	CL<br>CL	ND<br>ND	P<br>P
**Case 12**	*CLCNKB/CLCNKA*	1p36.13(16353804 -16374539)<br>1p36.13(16353804 -16374539)	exon 6-20 *CLCNKA*- exon 1-7 *CLCNKB* del<br>exon 6-20 *CLCNKA*- exon 1-7 *CLCNKB* del	NA<br>NA	CL<br>CL	ND<br>ND	P<br>P
**Case 13**	*SLC12A3*	c.626G>A<br>c.1390G>A	p.Arg209Gln<br>p.Ala464Thr	mat<br>pat	PL<br>PL	[6]<br>[7]	P<br>P
**Case 14**	*SLC12A3*	c.1930delC<br>c.2581C>T	p.Gln644Serfs*28<br>p.Arg861Cys	NA<br>NA	CL<br>PL	[8]<br>[9]	P<br>P
**Case 15**	*CLCNKB*	c.1783C>T<br>1p36.13(16.364.253-16.383.299)x1	p.Arg595*<br>entire gene deletion	mat<br>pat	CL<br>CL	[10]<br>[4]	P<br>P

**Case 16**	*SLC12A1*	c.768C>G<br>c.953C>A	p.Phe256Leu<br>p.Ala318Asp	NA<br>NA	PL<br>PL	ND<br>ND	LP<br>V
**Case 17**	*CFTR*	c.1001G>T<br>c.3909C>G	p.Arg334Leu<br>p.Asn1303Lys	pat<br>mat	PL<br>PL	[11]<br>[12]	P<br>P
**Case 18**	*SLC12A3*	c.1489A>T<br>c.3053G>A	p.Lys497*<br>p.Arg1018Gln	pat<br>mat	CL<br>PL	[6]<br>[12]	P<br>P
**Case 19**	*SLC12A3*	c.1928C>T<br>c.2221G>A	p.Pro643Leu<br>p.Gly741Arg	NA<br>NA	PL<br>PL	[6]<br>[13]	P<br>P
**Case 20**	*SLC12A3*	c.965-1_976delins12<br>16q13(56927331_56936430)x1	p.splicing aleration<br>exon20_24 del	pat<br>mat	CL<br>CL	[14]<br>[15]	P<br>P
**Case 21**	*CASR*	c.2528C>A	p.Ala843Glu	de novo	PL	[16]	P
**Case 23**	*SLC12A1*	15q21.1(48533711-48533796)<br>15q21.1(48533711-48533796)	exon 10 del<br>exon 10 del	mat<br>NA	CL<br>CL	ND<br>ND	P<br>P
**Case 24**	*KCNJ1*	c.133A>G<br>c.757G>C	p.Lys45Glu<br>p.Val253Leu	mat<br>pat	PL<br>PL	[17]<br>ND	LP<br>LP

**Case 26**	*SLC12A3*	c.1046C>T<br>c.1180+1G>T	p.Pro349Leu<br>p.spicing alteration	pat<br>mat	PL<br>CL	[13]<br>[18]	P<br>P
**Case 27**	*SLC12A3*	c.1046C>T<br>c.1180+1G>T	p.Pro349Leu<br>p.spicing alteration	pat<br>mat	PL<br>CL	[13]<br>[18]	LP<br>P
**Case 28**	*SLC12A3*	c.533C>T<br>c.625C>T	p.Ser178Leu<br>p.Arg209Trp	NA<br>NA	PL<br>PL	[6]<br>[13]	P<br>LP
**Case 29**	*SLC12A3*	c.1844C>T<br>c.1844C>T	p.Ser615Leu<br>p.Ser615Leu	NA<br>NA	PL<br>PL	[6]<br>[6]	P<br>P
**Case 30**	*SLC12A3*	c.20_21delCA<br>c.20_21delCA	p.Thr7Argfs*22<br>p.Thr7Argfs*22	mat<br>pat	CL<br>CL	[19]<br>[19]	P<br>P
**Case 31**	*SLC12A3*	c.1964G>A<br>c.2221G>A	p.Arg655His<br>p.Gly741Arg	NA<br>NA	PL<br>PL	[13]<br>[13]	P<br>P
**Case 32**	*SLC12A3*	c.2929C>T<br>16q13(56917504_56921771)x1	p.Arg977*<br>del exon 14-17	NA<br>NA	CL<br>CL	[13]<br>ND	P<br>P
**Case 36**	*SLC12A3*	c.1924C>G<br>c.1844C>T	p.Arg642Gly<br>p.Ser615Leu	mat<br>pat	PL<br>PL	[6]<br>[6]	P<br>LP

**Case 37**	*SLC12A3*	c.2581C>T<br>c.1180+1G>T	p.Arg861Cys<br>p.splicing alteration	NA<br>NA	PL<br>CL	[9]<br>[18]	P<br>P
**Case 38**	*SLC12A3*	c.2951+5G>A<br>c.2951+5G>A	p.splicing alteration<br>p.splicing alteration	NA<br>NA	CL<br>CL	ND<br>ND	P<br>P
**Case 39**	*SLC12A3*	c.2981G>A<br>c.20_21delCA	p.Cys994Tyr<br>p.Thr7Argfs*22	pat<br>mat	PL<br>CL	[7]<br>[19]	P<br>P
**Case 40**	*SLC12A3*	c.1180+1G>T<br>c.1180+1G>T	p.splicing alteration<br>p.splicing alteration	mat<br>pat	CL<br>CL	[18]<br>[18]	P<br>P
**Case 41**	*SLC12A3*	c.1180+1G>T<br>c.1180+1G>T	p.splicing alteration<br>p.splicing alteration	mat<br>pat	CL<br>CL	[18]<br>[18]	P<br>P
**Case 42**	*SLC12A3*	c.20_21delCA<br>c.237_238dupCC	p. Thr7Argfs*22<br>p.Arg80Profs*35	pat<br>mat	CL<br>CL	[19]<br>[20]	P<br>P
**Case 43**	*SLC12A3*	c.473G>A<br>c.1924C>G	p.Arg158Gln<br>p.Arg642Gly	pat<br>mat	PL<br>PL	[7]<br>[6]	P<br>P
**Case 44**	*SLC12A3*	c.992C>T<br>c.2682delG	p.Pro331Leu<br>p.Lys894fs *30	NA<br>pat	PL<br>CL	ND<br>ND	LP<br>P

**Case 45**	*SLC12A3*	c.1850A>G<br>c.1850A>G	p.Gln617Arg<br>p.Gln617Arg	mat<br>pat	PL<br>PL	[21]<br>[21]	LP<br>LP
**Case 46**	*SLC12A3*	c.378delC<br>c.378delC	p.Ser126Argfs*17<br>p.Ser126Argfs*17	mat<br>pat	CL<br>CL	ND<br>ND	P<br>P
**Case 47**	*SLC12A3*	c.1403C>G<br>c.2221G>A	p.Ser468Cys<br>p.Gly741Arg	pat<br>mat	PL<br>PL	ND<br>[13]	LP<br>P
**Case 48**	*SLC12A3*	c.237_238dupCC<br>c.2213T>G	p.Arg80Profs*35<br>p.Leu738Arg	NA<br>NA	CL<br>PL	[20]<br>[9]	P<br>P
**Case 49**	*SLC12A3*	c.179C>T<br>c.1124C>A	p.Thr60Met<br>p.Thr375Asn	pat<br>mat	PL<br>PL	[8]<br>[22]	P<br>LP
**Case 52**	*SLC12A3*	c.1709C>T<br>c.1373C>T	p.Ala570Val<br>p.Thr458Met	NA<br>NA	PL<br>PL	[23]<br>ND	LP<br>LP
**Case 53**	*SLC12A3*	c.1742T>A<br>c.3070G>A	p.Met581Lys<br>p.Val1024Met	NA<br>NA	PL<br>PL	[19]<br>[3]	P<br>P

**Case 54**	*SLC12A3*	c.1742T>A<br>c.3070G>A	p.Met581Lys<br>p.Val1024Met	NA<br>NA	PL<br>PL	[19]<br>[3]	P<br>P
**Case 55**	*SLC12A3*	c.1924C>G<br>c.2981G>A	p.Arg642Cys<br>p.Cys994Tyr	pat<br>mat	PL<br>PL	[24]<br>[7]	P<br>P
**Case 56**	*SLC12A3*	c.1924C>G<br>c.2981G>A	p.Arg642Cys<br>p.Cys994Tyr	pat<br>mat	PL<br>PL	[24]<br>[7]	P<br>P
**Case 57**	*CLCNKB*	c.1054C>G<br>c.1312C>T	p.Leu352Val<br>p.Arg438Cys	mat<br>de novo	PL<br>PL	ND<br>[4]	P<br>LP
**Case 58**	*SLC12A3*	c.1028T>A<br>c.2581C>T	p.Met343Lys<br>p.Arg861Cys	pat<br>mat	PL<br>PL	[3]<br>[9]	LP<br>P
**Case 61**	*SLC12A3*	c.1924C>T<br>c.1180+1G>T	p.Arg642Cys<br>p.splicing alteration	mat<br>pat	PL<br>CL	[24]<br>[18]	P<br>P
**Case 62**	*SLC12A3*	c.947G>T<br>c.1181G>A	p.Gly316Val<br>p.Gly394Asp	mat<br>pat	PL<br>PL	[7]<br>[25]	P<br>P
**Case 63**	*CLDN10*	c.200C>G<br>c.526G>T	p.Pro67Arg<br>p.Gly176Cys	NA<br>mat	PL<br>PL	ND<br>ND	LP<br>LP

ACMG, American College of Medical genetics and Genomics; CL, complete loss-of-function; PL, partial loss-of-function; ND, not described; P, pathogenic; LP, likely pathogenic; V, variant of unknown significance; mat, maternal; pat, paternal; NA, not available.

Table S 4. Summary of the results of WES.

Patient	Age at clinical diagnosis	Pre-WES diagnosis	Age at genetic diagnosis	Post-WES diagnosis	Gene	MIM number (#)
**Case 1**	2 months	BS	3 months	BS1	*SLC12A1*	601678
**Case 2**	At birth	BS	1 month	BS1	*SLC12A1*	601678
**Case 3**	12 years	BS	12 years	BS1	*SLC12A1*	601678
**Case 4**	At birth	BS	10 years	BS1	*SLC12A1*	601678
**Case 5**	At birth	BS	1 year	BS2	*KCNJ1*	241200
**Case 6**	At birth	BS	1 year	BS3	*CLCNKB*	607364
**Case 7**	At birth	BS	1 month	BS3	*CLCNKB*	607364
**Case 8**	6 months	BS	19 years	BS3	*CLCNKB*	607364
**Case 9**	4 months	BS	3 years	BS3	*CLCNKB*	607364
**Case 10**	At birth	BS	2 years	BS4a	*BSND*	602522
**Case 11**	7 years	BS	7 years	BS4b	*CLCNKA/CLCNKB*	613090
**Case 12**	2 months	BS	2 months	BS4b	*CLCNKA/CLCNKB*	613090
**Case 13**	5 years	BS	5 years	GS	*SLC12A3*	263800
**Case 14**	7 years	BS	34 years	GS	*SLC12A3*	263800
**Case 15**	3 months	BS	9 years	BS3	*CLCNKB*	607364
**Case 16**	3 years	BS	4 years	BS1	*SLC12A1*	601678
**Case 17**	4 months	BS	6 months	CYSTIC FIBROSIS	*CFTR*	219700
**Case 18**	2 years	BS	18 years	GS	*SLC12A3*	263800
**Case 19**	14 years	BS	14 years	GS	*SLC12A3*	263800
**Case 20**	2 years	BS	3 years	GS	*SLC12A3*	263800
**Case 21**	11 months	BS	1 year	HYPOC1	*CASR*	601198
**Case 22**	15 days	BS	8 years	-	-	-
**Case 23**	2 months	BS	19 years	BS1	*SLC12A1*	601678
**Case 24**	At birth	BS	9 years	BS2	*KCNJ1*	241200
**Case 25**	41 years	GS	58 years	-	-	-
**Case 26**	16 months	GS	5 years	GS	*SLC12A3*	263800
**Case 27**	6 years	GS	10 years	GS	*SLC12A3*	263800

**Case 28**	25 years	GS	58 years	GS	*SLC12A3*	263800
**Case 29**	7 years	GS	7 years	GS	*SLC12A3*	263800
**Case 30**	3 years	GS	28 years	GS	*SLC12A3*	263800
**Case 31**	50 years	GS	50 years	GS	*SLC12A3*	263800
**Case 32**	3 years	GS	3 years	GS	*SLC12A3*	263800
**Case 33**	18 years	GS	18 years	-	-	-
**Case 34**	22 years	GS	58 years	-	-	-
**Case 35**	54 years	GS	62 years	-	-	-
**Case 36**	26 years	GS	28 years	GS	*SLC12A3*	263800
**Case 37**	26 years	GS	41 years	GS	*SLC12A3*	263800
**Case 38**	3,5 years	GS	8 years	GS	*SLC12A3*	263800
**Case 39**	14 years	GS	14 years	GS	*SLC12A3*	263800
**Case 40**	6 years	GS	7 years	GS	*SLC12A3*	263800
**Case 41**	3 years	GS	4 years	GS	*SLC12A3*	263800
**Case 42**	18 years	GS	20 years	GS	*SLC12A3*	263800
**Case 43**	12 years	GS	13 years	GS	*SLC12A3*	263800
**Case 44**	10 years	GS	44 years	GS	*SLC12A3*	263800
**Case 45**	7 years	GS	8 years	GS	*SLC12A3*	263800
**Case 46**	7 years	GS	8 years	GS	*SLC12A3*	263800
**Case 47**	18 months	GS	20 years	GS	*SLC12A3*	263800
**Case 48**	34 years	GS	36 years	GS	*SLC12A3*	263800
**Case 49**	9 years	GS	11 years	GS	*SLC12A3*	263800
**Case 50**	26 years	GS	26 years	-	-	-
**Case 51**	50 years	GS	51 years	-	-	-
**Case 52**	54 years	GS	64 years	GS	*SLC12A3*	263800
**Case 53**	NA	GS	52 years	GS	*SLC12A3*	263800
**Case 54**	NA	GS	55 years	GS	*SLC12A3*	263800
**Case 55**	6 years	GS	6 years	GS	*SLC12A3*	263800
**Case 56**	5 years	GS	6 years	GS	*SLC12A3*	263800
**Case 57**	21 years	GS	27 years	BS3	*CLCNKB*	607364

**Case 58**	16 years	GS	16 years	GS	*SLC12A3*	263800
**Case 59**	39 years	GS	49 years	-	-	-
**Case 60**	38 years	GS	47 years	-	-	-
**Case 61**	6 years	GS	8 years	GS	*SLC12A3*	263800
**Case 62**	2 years	GS	11 years	GS	*SLC12A3*	263800
**Case 63**	22 years	GS	22 years	HELIX	*CLDN10*	617671

BS, Bartter Syndrome; GS, Gitelman Syndrome; BS1, Bartter syndrome type 1; BS2, Bartter syndrome type 2; BS3, Bartter syndrome type 3; BS4a, Bartter syndrome type 4a; BS4b, Bartter syndrome type 4b; HYPOC1, Hypocalcemia autosomal dominant 1*; HELIX, HELIX syndrome; NA, not available.*

Supplementary bibliography

1. Brochard, K.; Boyer, O.; Blanchard, A.; Loirat, C.; Niaudet, P.; Macher, M.-A.; Deschenes, G.; Bensman, A.; Decramer, S.; Cochat, P.; et al. Phenotype-Genotype Correlation in Antenatal and Neonatal Variants of Bartter Syndrome. *Nephrol. Dial. Transplant* **2009**, *24*, 1455–1464.
2. Puricelli, E.; Bettinelli, A.; Borsa, N.; Sironi, F.; Mattiello, C.; Tammaro, F.; Tedeschi, S.; Bianchetti, M.G.; Italian Collaborative Group for Bartter Syndrome Long-Term Follow-up of Patients with Bartter Syndrome Type I and II. *Nephrol. Dial. Transplant* **2010**, *25*, 2976–2981.
3. Ji, W.; Foo, J.N.; O'Roak, B.J.; Zhao, H.; Larson, M.G.; Simon, D.B.; Newton-Cheh, C.; Matthew W State; Levy, D.; Lifton, R.P. Rare Independent Mutations in Renal Salt Handling Genes Contribute to Blood Pressure Variation. *Nature Genetics* 2008, *40*, 592–599.
4. Simon, D.B.; Bindra, R.S.; Mansfield, T.A.; Nelson-Williams, C.; Mendonca, E.; Stone, R.; Schurman, S.; Nayir, A.; Alpay, H.; Bakkaloglu, A.; et al. Mutations in the Chloride Channel Gene, CLCNKB, Cause Bartter's Syndrome Type III. *Nat. Genet.* **1997**, *17*, 171–178.
5. Seys, E.; Andrini, O.; Keck, M.; Mansour-Hendili, L.; Courand, P.-Y.; Simian, C.; Deschenes, G.; Kwon, T.; Bertholet-Thomas, A.; Bobrie, G.; et al. Clinical and Genetic Spectrum of Bartter Syndrome Type 3. *J. Am. Soc. Nephrol.* **2017**, *28*, 2540–2552.
6. Cruz, D.N.; Shaer, A.J.; Bia, M.J.; Lifton, R.P.; Simon, D.B. Gitelman's Syndrome Revisited: An Evaluation of Symptoms and Health-Related Quality of Life. *Kidney International* 2001, *59*, 710–717.
7. Syrén, M.-L.; Tedeschi, S.; Cesareo, L.; Bellantuono, R.; Colussi, G.; Procaccio, M.; Alì, A.; Domenici, R.; Malberti, F.; Sprocati, M.; et al. Identification of Fifteen Novel Mutations in the SLC12A3 Gene Encoding the Na-Cl Co-Transporter in Italian Patients with Gitelman Syndrome. *Hum. Mutat.* **2002**, *20*, 78.
8. Maki, N.; Komatsuda, A.; Wakui, H.; Ohtani, H.; Kigawa, A.; Aiba, N.; Hamai, K.; Motegi, M.; Yamaguchi, A.; Imai, H.; et al. Four Novel Mutations in the Thiazide-Sensitive Na-Cl Co-Transporter Gene in Japanese Patients with Gitelman's Syndrome. *Nephrology Dialysis Transplantation* 2004, *19*, 1761–1766.
9. Lemmink, H.H.; Knoers, N.V.; Károlyi, L.; van Dijk, H.; Niaudet, P.; Antignac, C.; Guay-Woodford, L.M.; Goodyer, P.R.; Carel, J.C.; Hermes, A.; et al. Novel Mutations in the Thiazide-Sensitive NaCl Cotransporter Gene in Patients with Gitelman Syndrome with Predominant Localization to the C-Terminal Domain. *Kidney Int.* **1998**, *54*, 720–730.
10. Bettinelli, A.; Borsa, N.; Bellantuono, R.; Syrèn, M.-L.; Calabrese, R.; Edefonti, A.; Komninos, J.; Santostefano, M.; Beccaria, L.; Pela, I.; et al. Patients with Biallelic Mutations in the Chloride Channel Gene CLCNKB: Long-Term Management and Outcome. *Am. J. Kidney Dis.* **2007**, *49*, 91–98.
11. Dörk, T.; Dworniczak, B.; Aulehla-Scholz, C.; Wieczorek, D.; Böhm, I.; Mayerova, A.; Seydewitz, H.H.; Nieschlag, E.; Meschede, D.; Horst, J.; et al. Distinct Spectrum of CFTR Gene Mutations in Congenital Absence of Vas Deferens. *Hum. Genet.* **1997**, *100*, 365–377.
12. Yasujima M. [Clinical significance of thiazide-sensitive Na-Cl cotransporter gene by mutational analysis]. *Rinsho Byori* **2007**, *55*, 338–343.
13. Simon, D.B.; Nelson-Williams, C.; Bia, M.J.; Ellison, D.; Karet, F.E.; Molina, A.M.; Vaara, I.; Iwata, F.; Cushner, H.M.; Koolen, M.; et al. Gitelman's Variant of Barter's Syndrome, Inherited Hypokalaemic Alkalosis, Is Caused by Mutations in the Thiazide-Sensitive Na–Cl Cotransporter. *Nature Genetics* 1996, *12*, 24–30.
14. Shao, L.; Liu, L.; Miao, Z.; Ren, H.; Wang, W.; Lang, Y.; Yue, S.; Chen, N. A Novel SLC12A3 Splicing Mutation Skipping of Two Exons and Preliminary Screening for Alternative Splice Variants in Human Kidney. *Am. J. Nephrol.* **2008**, *28*, 900–907.
15. Shen, Q.; Chen, J.; Yu, M.; Lin, Z.; Nan, X.; Dong, B.; Fang, X.; Chen, J.; Ding, G.; Zhang, A.; et al. Multi-Centre Study of the Clinical Features and Gene Variant Spectrum of Gitelman Syndrome in Chinese Children. *Clin. Genet.* **2021**, *99*, 558–564.
16. Zhao, X.M.; Hauache, O.; Goldsmith, P.K.; Collins, R.; Spiegel, A.M. A Missense Mutation in the Seventh Transmembrane Domain Constitutively Activates the Human Ca2+ Receptor. *FEBS Lett.* **1999**, *448*, 180–184.
17. Schnakenburg, C. von; Frankenschmidt, A.; Neumann, J.; Häffner, K.; Jeck, N.; Pohl, M.

[Polyhydramnios, prematurity, dystrophy, polyuria, constipation, nephrocalcinosis and renal tumor: presentation of a classic tubulopathy]. *Klin. Padiatr.* **2008**, *220*, 24–25.
18. Coto, E.; Rodriguez, J.; Jeck, N.; Alvarez, V.; Stone, R.; Loris, C.; Rodriguez, L.M.; Fischbach, M.; Seyberth, H.W.; Santos, F. A New Mutation (intron 9 +1 G>T) in the SLC12A3 Gene Is Linked to Gitelman Syndrome in Gypsies. *Kidney Int.* **2004**, *65*, 25–29.
19. Colussi, G.; Bettinelli, A.; Tedeschi, S.; De Ferrari, M.E.; Syrén, M.L.; Borsa, N.; Mattiello, C.; Casari, G.; Bianchetti, M.G. A Thiazide Test for the Diagnosis of Renal Tubular Hypokalemic Disorders. *Clin. J. Am. Soc. Nephrol.* **2007**, *2*, 454–460.
20. Mastroianni, N.; Bettinelli, A.; Bianchetti, M.; Colussi, G.; De Fusco, M.; Sereni, F.; Ballabio, A.; Casari, G. Novel Molecular Variants of the Na-Cl Cotransporter Gene Are Responsible for Gitelman Syndrome. *Am. J. Hum. Genet.* **1996**, *59*, 1019–1026.
21. Zeng, Y.; Li, P.; Fang, S.; Wu, C.; Zhang, Y.; Lin, X.; Guan, M. Genetic Analysis of SLC12A3 Gene in Chinese Patients with Gitelman Syndrome. *Med. Sci. Monit.* **2019**, *25*, 5942–5952.
22. Vargas-Poussou, R.; Dahan, K.; Kahila, D.; Venisse, A.; Riveira-Munoz, E.; Debaix, H.; Grisart, B.; Bridoux, F.; Unwin, R.; Moulin, B.; et al. Spectrum of Mutations in Gitelman Syndrome. *J. Am. Soc. Nephrol.* **2011**, *22*, 693–703.
23. Yoo, T.-H.; Lee, S.-H.; Yoon, K.; Baek, H.; Chung, J.-H.; Lee, T.; Ihm, C.; Kim, M. Identification of Novel Mutations in Na-Cl Cotransporter Gene in a Korean Patient with Atypical Gitelman's Syndrome. *Am. J. Kidney Dis.* **2003**, *42*, E11–E16.
24. Yahata, K.; Tanaka, I.; Kotani, M.; Mukoyama, M.; Ogawa, Y.; Goto, M.; Nakagawa, M.; Sugawara, A.; Tanaka, K.; Shimatsu, A.; et al. Identification of a Novel R642C Mutation in Na/Cl Cotransporter with Gitelman's Syndrome. *Am. J. Kidney Dis.* **1999**, *34*, 845–853.
25. Ravarotto, V.; Loffing, J.; Loffing-Cueni, D.; Heidemeyer, M.; Pagnin, E.; Calò, L.A.; Rossi, G.P. Gitelman's Syndrome: Characterization of a Novel c.1181G>A Point Mutation and Functional Classification of the Known Mutations. *Hypertens. Res.* **2018**, *41*, 578–588.
